# Reproducibility of physical activity recall over fifteen years: longitudinal evidence from the CARDIA study

**DOI:** 10.1186/1471-2458-13-180

**Published:** 2013-02-28

**Authors:** Ashley Wilder Smith, Kathleen A Cronin, Heather Bowles, Gordon Willis, David R Jacobs, Rachel Ballard-Barbash, Richard P Troiano

**Affiliations:** 1 Applied Research Program, Division of Cancer Control and Population Sciences, National Cancer Institute, Bethesda, MD, USA; 2 Surveillance Research Program, Division of Cancer Control and Population Sciences, National Cancer Institute, Bethesda, MD, USA; 3 Division of Epidemiology and Community Health, School of Public Health, University of Minnesota, Minneapolis, MN, USA; 4 Department of Nutrition, School of Medicine, University of Oslo, Oslo, Norway; 5 Outcomes Research Branch, Applied Research Program, National Cancer Institute, Executive Plaza North, Room 4005, 6130 Executive Boulevard, MSC 7344, Bethesda, MD 20892-7344, USA

## Abstract

**Background:**

To examine the benefits of physical activity (PA) on diseases with a long developmental period, it is important to determine reliability of long-term PA recall.

**Methods:**

We investigated 15-year reproducibility of PA recall. Participants were 3605 White and African-American adults in the Coronary Artery Risk Development in Young Adults study, aged 33–45 at the time of recall assessment. Categorical questions assessed PA before and during high school (HS) and overall PA level at Baseline, with the same timeframes recalled 15 years later. Moderate- and vigorous-intensity scores were calculated from reported months of participation in specific activities.

**Results:**

HS PA recall had higher reproducibility than overall PA recall (weighted kappa = 0.43 vs. 0.21). Correlations between 15-year recall and Baseline reports of PA were r = 0.29 for moderate-intensity scores, and r = 0.50 for vigorous-intensity. Recall of vigorous activities had higher reproducibility than moderate-intensity activities. Regardless of number of months originally reported for specific activities, most participants recalled either no activity or activity during all 12 months.

**Conclusion:**

PA recall from the distant past is moderately reproducible, but poor at the individual level, among young and middle aged adults.

## Background

Abundant research demonstrates inverse associations between physical activity (PA) level and chronic disease risks, including cardiovascular disease, diabetes, hypertension and certain cancers [1]. Although clinical manifestations of diseases such as breast cancer do not typically become evident until middle age or older, the pathological etiology may be associated with PA during young adulthood or childhood [2]. It is therefore important to reliably and validly assess participation in PA over the lifespan to fully examine relationships between PA and chronic disease development.

In epidemiological studies that follow participants from late adulthood, PA participation over the lifespan may be assessed by recall of historical PA. Historical PA is defined as activity engaged in more than one year before the assessment [3-6]. Studies have correlated historical PA with: cardiorespiratory fitness and health markers documented in medical records [3]; personal reports to a physician at a prior point in time [7], and current health markers [8]. However, time constraints and lack of criterion measures are challenges to assessing the validity of historical PA recall.

This analysis extends previous research from the Coronary Artery Risk Development in Young Adults (CARDIA) study [9] by analyzing the reproducibility of historical PA recall for the entire CARDIA sample, and examining differences in reproducibility by age, sex, and race subgroups. The time period between historical PA recalls was 15 years, which may approximate a period of time relevant to studies of cancer and other chronic diseases. Specific objectives were to: (1) examine the reproducibility of reporting PA occurring during adolescence and young adulthood; (2) assess reproducibility differences for reporting PA frequency, type, and intensity; (3) identify demographic characteristics associated with reproducibility differences.

## Methods

### Study participants

CARDIA is a longitudinal, population-based cohort study examining determinants of coronary artery disease risk factors in young adults. The cohort included 5115 adults. There were 5045 males and females aged 18–30 years (those <18 were excluded), with approximately equal distribution by race (black and white), sex, education (high school or less, and more than high school) and age (18–24 and 25–30). Baseline assessment occurred in Year 0 (1985–1986), with follow-up examinations conducted in Years 2, 5, 7, 10, 15, and 20. Details on CARDIA have been summarized previously [10]. Participants provided written informed consent at each examination, and institutional review boards at each field center (University of Alabama at Birmingham; Northwestern University; University of Minnesota; and Oakland, California Kaiser Permanente) and at the coordinating center (University of Alabama at Birmingham) approved the study annually. The analytic sample includes 3605 participants who provided complete demographic and PA data at Year 0 and Year 15.

### Physical activity measurement

PA outcomes included categorical ratings and continuous scores. Categorical questions assessed PA before and during high school (HS) and overall PA level during the year before the interview. Moderate- and vigorous-intensity scores were calculated from reported months of participation in specific activities. PA information was obtained for each participant at different time points: (a) Baseline PA (at Year 0), (b) Current PA (at Year 15), and (c) Historical PA (Recall of Baseline at Year 15).

#### Assessment of baseline physical activity at year 0

During the Year 0 exam, participants reported their overall PA level on a 5-point ordinal scale (‘inactive’ to ‘very active’) at specific times in life: (a) before HS, (b) during HS, and (c) during the 12 months before the exam. Respondents also reported their Baseline PA participation in thirteen specific moderate- and vigorous-intensity leisure time activities over the prior year: whether they had engaged in each activity (yes/no), for how many months (0–12), and how many of the months activities were performed for an activity-specific long duration. (See Sample Item for example survey questions on running.) Based on the CARDIA PA assessment protocol [11], questionnaire responses for the specific activities were also combined to form continuous moderate and vigorous-intensity leisure Baseline PA scores. Moderate, vigorous, and total scores were calculated as the sum of each activity MET level × months of infrequent activity plus 3 × months of frequent activity.

Sample item from physical activity questionnaire.

1. Did you jog or run in the 12 months before your first CARDIA exam for at least one hour total time in any month? For instance, you might have done three 20-minute sessions in the month.

_____ No

_____ Yes

2. How many months did you do this activity?

_____ months

3. How many of these months did you do this activity for at least 2 hours per week?

_____ months

#### Assessment of current and historical physical activity at year 15

In the Year 15 examination (conducted in 2000–2001) participants first reported their current PA by reporting moderate- and vigorous-intensity activities over the prior year using the same interview-administered instrument as in the Year 0 exam. After reporting their current PA, the interviewer provided prompts to help participants focus on the time period relevant to their Year 0 exam. First, they were primed with information about the month and year of their Year 0 exam visit, and about both their age and the President of the United States at that time. Additionally, each study center could mention a local event that had occurred at the time of the Year 0 exam. Participants were also asked questions about their living situation, job, school, marital status and whether they had children at that time. Following these reminders, and using the same questionnaire administered at Year 0, participants recalled their historical PA at Baseline by reporting their participation in the thirteen leisure time activities, including the number of months they had participated in each activity. They also reported their overall PA level before HS, during HS, and during the 12 months before the Year 0 exam, using the same 5-point ordinal scale used at Year 0 [12].

### Data analysis

PA at Baseline (Year 0), Current (past year at Year 15) and Historical (Recall of Baseline at Year 15) data enabled several levels of analysis: (1) whether participants could consistently classify their activity levels at particular times in their lives (e.g. HS); (2) whether they could consistently report participation in a particular activity (yes/no) in the past; (3) whether they could consistently report the number of months they participated in a particular activity in the past; and (4) whether demographic characteristics explained differences between Baseline and Historical PA reports.

We compared the distribution of demographics, body mass index (BMI), and PA score for the analytic sample at Year 0 and Year 15 with the entire enrolled CARDIA sample. We then examined reproducibility of Baseline and Historical ordinal categories of past year, HS and pre-HS activity levels with weighted kappa statistics and percent agreement. To examine whether participants tended to report Historical PA that reflected Current PA, agreement between Historical PA and Current PA was tested with percent agreement and weighted kappa statistics. Reproducibility of activity type was examined with percent agreement and kappa statistics. To explore whether participants could accurately reproduce the number of months they participated in a particular activity, we plotted the number of months they recalled doing specific activities against the number of months reported at Baseline. The number of months was collapsed into 3-month ranges to simplify presentation. For participants who reported that they did not engage in a particular activity, number of months was coded as 0. Subsequent analyses examined Baseline and Historical leisure PA scores for moderate- and vigorous-intensity activities using Spearman correlations. Similar to the categorical reports, we also examined correlations between Current PA scores and Historical PA scores. To determine correlates of reproducibility, we examined differences between Baseline PA and Historical PA scores in regression models. Covariates included Baseline activity levels (in quartiles), demographic and behavioral characteristics, and BMI. Discrepancies were examined by subtracting Baseline moderate- and vigorous-intensity activity scores from Historical scores; thus, positive scores indicate over-reporting at Year 15 compared to Year 0 and negative scores indicate under-reporting at Year 15 compared to Year 0.

## Results

Table 1 summarizes sample characteristics. The distribution of sex, age, education, BMI, and PA scores was similar between the analytic sample at Year 0 and the entire enrolled CARDIA sample; there were slightly more white participants in the analytic sample than in the enrolled sample. By Year 15, educational attainment, overweight, and obesity increased and PA scores decreased in the analytic sample. PA scores at Year 15 were lower than Baseline PA scores for all race and sex groups.

**Table 1:** Demographic characteristics of the CARDIA sample at Year 0 and the analytic sample at year 0 and year 15

	**CARDIA sample**	**Analytic sample**	
	At Year 0	At Year 0	At Year 15
	1985-1986	1985-1986	2000-2001
Sample Size	5045	3565	3565
**Sex**			
Male	2293 (45.5%)	1573 (44.1%)	
Female	2752 (54.5%)	1992 (55.9%)	
**Race**			
White	2454 (48.6%)	1893 (53.1%)	
Black	2591 (51.4%)	1672 (46.9%)	
**Age at Year 0**			
18-24 years	2266 (44.9%)	1495 (41.9%)	
25-30 years	2779 (55.1%)	2070 (58.1%)	
**Education**			
High School or Less	1989 (39.4%)	1262 (35.4%)	815 (23.0%)
Some College	1677 (33.2%)	1194 (33.5%)	1107 (31.1%)
College Graduate	1379 (27.3%)	1109 (31.1%)	1639 (45.9%)
Missing			4
**Body Mass Index**			
Normal weight (<25 kg/m^2^)	3297 (65.3%)	2328 (65.3%)	1198 (33.6%)
Overweight (25–29 kg/m^2^)	1158 (23.0%)	827 (23.2%)	1165 (32.7%)
Obese (≥30 kg/m^2^)	590 (11.7%)	410 (11.5%)	1202 (33.7%)
**Physical Activity Score**^**a,b**^			
Black Males	474 (272, 726)	444 (251, 708)	356 (176, 622)
Black Females	228 (103, 396)	220 (100, 388)	173 (70, 327)
White Males	461 (288, 672)	468 (290, 684)	372 (208, 570)
White Females	351 (208, 543)	364 (214, 548)	278 (137, 454)

^a^ CARDIA Physical Activity Score = sum (for moderate/vigorous/total) MET level × months of infrequent activity plus 3 × months of frequent activity.

^b^ Data are median (25^th^, 75^th^ percentiles).

Table 2 presents 15-Year reproducibility of PA levels in adolescence and early adulthood. Weighted kappa and percent agreement indicated consistency between Baseline and Historical PA levels during HS, and lower consistency for PA engaged in either before HS or during the year before Baseline. To examine whether PA recall was influenced by current PA level, Historical PA category was compared to Current PA category. Kappa statistics and percentage agreement for Historical and Current PA categories were low, indicating that Current PA category was less associated with Historical PA category than was Baseline PA.

**Table 2:** Fifteen-year reproducibility of self-rated physical activity levels in adolescence and early adulthood between baseline and historical recall (n = 3,565)

**Self-rated physical activity**	**Baseline physical activity**^**a**^	**Historical physical activity**^**a**^	**Current physical activity**^**a**^	**Percent agreement**	**Weighted kappa**
**Before high school**					
Inactive	145 (4.1%)	87 (2.4%)		45.4%	0.32
Somewhat Active	264 (7.4%)	229 (6.4%)			
Moderately Active	1103 (30.9%)	904 (25.4%)			
Active	722 (20.3%)	657 (18.4%)			
Very active	1330 (37.3%)	1687 (47.3%)			
Missing	1	1			
**During high school**					
Inactive	93 (2.6%)	58 (1.6%)		51.4%	0.43
Somewhat Active	266 (7.5%)	242 (6.8%)			
Moderately active	1021 (28.6%)	804 (22.6%)			
Active	816 (22.9%)	729 (20.4%)			
Very active	1369 (38.4%)	1732 (48.6%)			
**At baseline**					
Inactive	244 (6.9%)	82 (2.3%)	228 (6.4%)	32.3% ^b^	0.21 ^b^
Somewhat Active	517 (14.5%)	228 (6.4%)	607 (17.0%)	27.8% ^c^	0.12 ^c^
Moderately Active	1377 (38.6%)	951 (26.7%)	1581 (44.4%)		
Active	792 (22.2%)	870 (24.4%)	614 (17.2%)		
Very active	633 (17.8%)	1432 (40.2%)	534 (15.0%)		
Missing	2	2	1		

^a^ Baseline Physical Activity = Activity reported at Year 0; Historical Physical Activity = Recalled Baseline Activity reported at Year 15; Current Physical Activity = Activity reported at Year 15.

^b^ Percent agreement and weighted kappa for Baseline and Historical Physical Activity;

^c^ Percent agreement and weighted kappa for Historical and Current Physical Activity.

Fifteen-year reproducibility of reported types of activities was higher for vigorous-intensity activities such as sports and running, and for activities with very low participation rates such as bowling or golf, than for more moderate activities (Table 3). For all activities, differences between Baseline and Historical recall indicated that respondents were more likely to omit activities that were reported in Year 0 than to report activities that were not originally reported, illustrated by the consistently higher numbers in the “Yes/No column” than the No/Yes column.

**Table 3:** 15-year reproducibility of reported types of activities between baseline and historical recall (n = 3,565)

	Baseline recall/Historical recall^*^				**Percent agreement**	**Kappa score**
	**No/No**	**Yes/Yes**	**No/Yes**	**Yes/No**		
**Moderate-intensity activities**						
Home exercise / Calisthenics	1408	704	304	1149	59%	.20
Non-strenuous sports	790	1395	527	853	61%	.21
Walk or hike	423	2035	397	710	69%	.23
Bowl or golf	2081	539	346	599	73%	.35
Gardening / Home maintenance	1462	987	315	801	69%	.37
**Vigorous-intensity activities**						
Bike	1353	939	479	794	64%	.28
Swim	2061	481	364	659	71%	.29
Home activity (shoveling, weight lifting)	1167	1144	341	913	65%	.31
Exercise/Dance class	1375	975	441	774	66%	.32
Job activity (lifting, carrying)	1738	736	369	722	69%	.34
Run	1261	1234	359	711	70%	.41
Racket sports	2320	496	313	436	79%	.43
Strenuous sports	1585	1071	358	551	74%	.48

*Year 0/Year 15.

**Figure 1:**
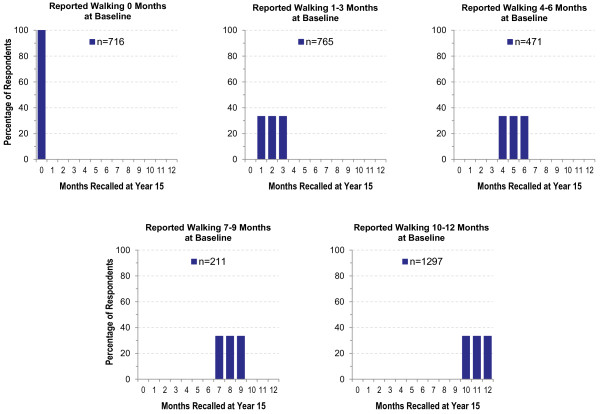
“Ideal” number of recalled months of activity if historical recall was perfect.

**Figure 2:**
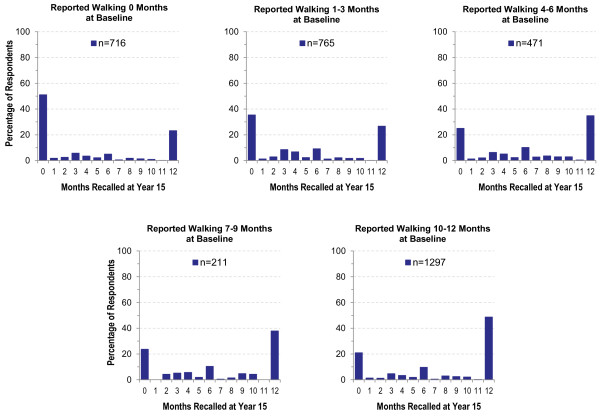
Actual months of reported walking at baseline compared to historical recall at year 15.

To explore 15-year reproducibility of reported months of PA participation we plotted the distribution of Historical months by number of months reported at Baseline for each activity. Figure 1 depicts ideal response distributions if participants are able to accurately recall their reported Baseline activity. These idealized data show 100% of those who originally reported no activity (0 months at Baseline) recalling 0 months. For those who reported 4, 5, or 6 months at Baseline, ideal recall is distributed across 4–6 months of activity, with no recalls of other than 4, 5, or 6 months. In contrast, Figure 2 depicts the plots of actual data for months of walking. An interesting pattern emerged: regardless of the number of months reported at Baseline, the majority of participants recalled doing either no activity (0 months) or activity during all 12 months, with some suggestion of clustering at 6 months. We observed a similar pattern for other moderate- and vigorous-intensity activities (data not shown).

**Figure 3:**
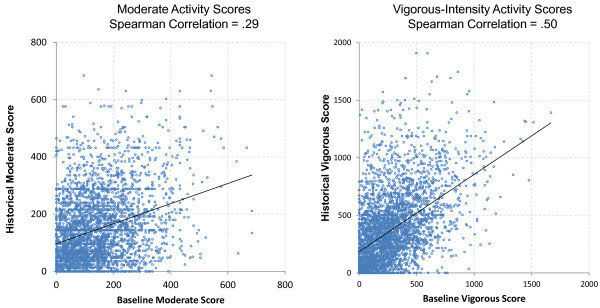
Association of baseline and historical moderate- and vigorous-intensity activity scores.

Examination of moderate- and vigorous-intensity PA scores reproducibility indicated a modest Spearman correlation between Baseline and Historical moderate-intensity scores (*r* = 0.29). The correlation for vigorous-intensity scores was stronger (*r* = 0.50). However, the spread of scores was extensive (see Figure 3), and represents a substantial amount of both under-and over-reporting at the individual level. Historical scores were modestly associated with Current scores for both moderate-intensity (*r* = 0.36) and vigorous-intensity scores (*r* = 0.37).

Finally, we applied multiple regression analysis to identify sample characteristics that may explain the differences between Baseline and Historical moderate- and vigorous-intensity scores. Both regression models were significant: model statistics for moderate-intensity activity recall were: *F* = 76.60, *R*^*2*^ = 0.26, *p* < 0.0001 and for vigorous-intensity activity recall were: *F* = 34.35, *R*^*2*^ = 0.14, *p* < 0.0001. Factors associated with the adjusted mean differences between PA scores reported at Baseline and those recalled Historically, are presented in Table 4. Overall, reported differences were larger and more likely to be positive (reflective of over-reporting) for vigorous than for moderate activity. Baseline PA was a significant predictor, in that the lower the Baseline PA level, the more likely participants were to over-report their Historical PA. For moderate intensity, the pattern of differences appeared to show regression to the mean, with the lower scores at Baseline showing over-reporting and higher scores showing under-reporting. For vigorous-intensity scores, participants were more likely to over-report, with the exception of the most active individuals. Regression analysis also indicated significant associations between several demographic variables and the difference in PA recalled scores, including interactions and main effects of sex and race, as well as education and race. Men over-reported vigorous-intensity Baseline levels to a greater extent than women; black men over-reported their activity levels to the greatest extent. Among black participants, higher education was associated with greater over-estimation. BMI level was also a significant predictor of moderate-intensity difference scores, with over-reporting of Historical PA increasing as BMI increased. Although there were several significant predictors in our models, associations between PA recall discrepancy and both season of the year and susceptibility to social desirability (measured by the Marlowe-Crowne Social Desirability Scale) [13] were explored, but neither was found to be statistically significant (data not shown). Overall, the regression models explained little of the variance in the difference scores for either moderate-intensity (R^2^ = 0.26) or vigorous-intensity scores (R^2^ = 0.14).

**Table 4:** Factors associated with adjusted mean differences between physical activity scores reported at baseline and recalled historically, stratified by intensity (n = 3,561)

	**Moderate-intensity activity score difference**	**Vigorous-intensity activity score difference**
	**Least squares mean**^**b**^	**Least squares mean**^**b**^
Baseline physical activity score^a^		
1^st^ quartile	78.9	191.1
2^nd^ quartile	50.8	165.2
3^rd^ quartile	−8.6	96.4
4^th^ quartile	−92.8	−19.8
Sex^c^		
Male	16.0	170.1
Female	−1.8	46.4
Race^c^		
White	−2.1	61.6
Black	16.3	154.9
Age at Year 0		
18-24 years	6.9	103.4
25-30 years	7.2	113.0
Education at Year 15^d^		
High school or Less	0.3	99.6
Some College	3.9	103.6
College Graduate	17.2	121.5
Body Mass Index at Year 15^d^		
Normal weight (<25 kg/m^2^)	3.3	97.9
Overweight (25–29 kg/m^2^)	3.5	115.7
Obese (≥30 kg/m^2^)	14.4	111.1
Sex & Race Interaction^c^		
Black Males	31.4	242.6
Black Females	1.3	67.2
White Males	0.6	97.6
White Females	−4.8	25.6
Race & Education Interaction^c^		
Black, High School or Less	−1.3	122.1
Black, Some College	17.0	165.4
Black, College Graduate	33.3	177.2
White, High School or Less	1.9	77.1
White, Some College	−9.1	41.8
White, College Graduate	0.8	65.8
PA Level & Age at Year 0 Interaction^c^		
1^st^ quartile, 18–25 years	85.1	200.2
1^st^ quartile, 26–34 years	70.7	182.0
2^nd^ quartile, 18–25 years	57.2	166.1
2^nd^ quartile, 26–34 years	44.6	164.3
3^rd^ quartile, 18–25 years	−14.9	108.4
3^rd^ quartile, 26–34 years	−2.4	84.4
4^th^ quartile, 18–25 years	−99.7	−61.0
4^th^ quartile, 26–34 years	−86.0	21.4

^a^ CARDIA Physical Activity Score = sum (for moderate or vigorous) MET level x months of infrequent activity plus 3 x months of frequent activity.

^b^ Difference scores = Recall – Baseline; positive least squares means represent over-reporting.

^c^*p* < 0.05.

^d^*p* < 0.05 for moderate-intensity activity score only.

## Discussion

Accurate and reliable PA assessment is essential for epidemiologists, exercise scientists, clinicians, and behavioral researchers. Recently, objective measures of PA, such as accelerometers, have dominated the public health literature [1]. Objective devices may assess current behavior well; however they cannot provide information about PA from the distant past. To understand the influence of historical PA on chronic disease risk, it is important to know whether PA can be reasonably recalled over the long-term and to assess demographic, social, and behavioral factors that may affect recall.

Results of our comparison of PA recalled over 15 years to original reports varied by both the type of survey question and the type of information obtained. Respondents were able to reproduce categorical ratings of overall PA level (e.g., ‘very active’) reasonably well, particularly for a well-defined and significant time such as during HS (percent agreement >50% and Kappa = 0.43). As displayed in Table 2, percent agreement and Kappa were more modest for periods that were likely to be less memorable, such as before HS or the year before entry into the CARDIA study. It is also possible that HS activity was recalled well because it is a period that may be recalled and reported more often than less well-defined periods. Agreement for reports of whether respondents participated in specific activities was also reasonably reliable (see Table 3), particularly for vigorous-intensity activities (percent agreement 64-79% and Kappa 0.28-0.48). However, in some cases, high agreement may be due to the low participation rate for these activities (e.g., bowling/golf), so that one agreement category (No/No) is very large. As a result, it is not possible to determine which specific activities can be recalled best. When more quantification was involved, such as estimating the number of participation months for an activity, agreement was negatively affected by a combination of a propensity to exclude activities in the Historical report, as well as response clustering at 0 and 12 months of participation, as shown in Figures 1 and 2. It appeared that long-term recall led to a loss of time resolution, with a tendency toward all-or-none estimation, and perhaps some “splitting the difference” by estimating a value of 6 months.

In general, our data showed that PA recall consistency over fifteen years among young and middle-aged adults was generally modest, but comparable to studies of similar and longer duration [14-19]. However, an important observation is that even when overall agreement was reasonably good for long term recall studies of this type -- such as the correlation of 0.50 for the vigorous activity score -- error at the individual level was quite large. As shown in Figure 3, for a vigorous-intensity score of 500 at Baseline, the Historical scores ranged from 0 to 1500. This substantial error at the individual level will likely reduce the researcher’s ability to detect relationships between historical activity and outcomes within individuals. However, on the positive side, in contrast to Falkner et al. [20], historical recall in this study appeared to reflect actual recall, rather than current activity. With the exception of the Moderate Activity Score, for which agreement of Historical scores with Current score was similar to agreement with Baseline score, Historical reports were more similar to Baseline than they were to Current reports of PA.

The large CARDIA cohort allowed examination of demographic and other predictors of reporting discrepancy. Generally, the Historical score was higher than Baseline, particularly for vigorous-intensity scores. These results indicate over-reporting of recalled physical activity and are consistent with previous studies [16,18]. Demographic characteristics including BMI, race and education were significantly associated with discrepancies in recall. A race by education interaction reflected the unexpected finding that over-reporting increased with higher levels of education among black participants. Our results also indicated an interaction of sex and race with the Baseline activity level that highlighted over-reporting by men, particularly black men. These results indicate that demographic factors need to be taken into consideration when pursuing studies of physical activity recall, and specific examination by subgroups should be considered. However, in our study, the recall discrepancy was also a function of Baseline activity scores, with the most active participants at Baseline likely to produce Historical activity scores lower than Baseline. Because true activity at the recalled time period is usually not available, it may be difficult to account for this source of error in studies that use recalled activity.

Overall, the current study adds to a growing body of research on long-term PA recall. These studies are important, as researchers increasingly use historical and lifetime measures to examine exposure to PA over the life course in relation to health outcomes [5,6,8,16]. A major limitation of methodological studies of long-term recall PA instruments is that validity can rarely be established due to the lack of criterion measures at the period(s) being reported. That leaves reliability or reproducibility of reports as the primary indicator of instrument quality. Reliability has generally not been examined relative to the actual period of interest. Many studies look at reproducibility of lifetime reports over 3–10 week periods [4,8,21], or up to one year [22]. These studies show that the reproducibility of self-reported lifetime activity recalled over short periods range from *r* = 0.53 to 0.85. Studies of specific activities recalled over longer periods (10–36 years) have shown weaker associations; correlations and kappa statistics range from 0.09-0.52 [14-19]. However, recall accuracy may have been affected by participants’ age, which varied from middle to older age.

This study has several notable strengths. Participants recalled activity over a long period of time (15 years) with the same instrument that was originally used. Other studies have used different instruments at two points in time, which has made interpretation and comparison of findings difficult [19]. The CARDIA cohort provided a large and diverse sample that allowed examination of factors related to the difference between Historical and Baseline reports. The current study focused on early adulthood and included questions about activity during adolescence. The Historical recall in CARDIA provides relevant data for studies of PA exposure in early life and later health outcomes.

There were also limitations to our study. We cannot determine whether PA recall reflected what participants were actually doing at the time of the Baseline exam, but rather what they reported doing. There are also potential limitations of generalizability. This study relied upon a single questionnaire in a cohort of black and white young and middle aged adults. Long term reproducibility is, at least in part, a function of the reliability of the questionnaire. The CARDIA questionnaire has been shown to have good reliability over two week retest (r ~ 0.80) [11]. It is not clear how well our results will generalize to other ethnic groups, older age groups, or to studies that use different questionnaires. Additionally, we cannot estimate the effect of participant dropout from CARDIA between Years 0 and 15.

However, our results provide an important step in understanding historical PA recall and have implications for future studies. For example, for investigators assembling retrospective cohort studies in which PA is used as an exposure variable, our data suggest that individuals do well at classifying their activity level with categorical questions, particularly for memorable life periods. Categorical responses can be important indicators; for example, a five-category single-item general health question has been shown to be related to health outcomes [23]. Seeking more quantitative precision in Historical recalls, on the other hand, may not be productive; our data showed that there was not much precision in participants’ ability to recall the amount of time over the course of a year for a given activity. These results provide important information when considering the kinds of questions that may be reasonably asked. In future research, investigators may want to consider whether the additional participant burden of asking about details regarding specific components of activity such as duration and frequency adds predictive value.

## Conclusions

The current study was able to expand on previous research by examining different components of PA recall. This study characterized novel, systematic patterns, such as the clustering of number of months of the year of reported PA. It also showed that participants were better able to recall vigorous-intensity activities, and could accurately reproduce their activity levels during salient times of life, such as HS. Overall, these data suggest that historical PA recall over 15 years is only modestly reproducible and poor at the individual level among young and middle aged adults. Researchers should consider these limitations when undertaking studies that require assessment of PA in the distant past.

## Competing interests

The authors declare that they have no competing interest.

## Authors’ contributions

AWS, RPT: Study conceptualization and project oversight; data analysis and interpretation; manuscript drafting and revision. KAC, HB: Data analysis and interpretation; manuscript drafting and revision. GW, DJ: Data interpretation; manuscript drafting and revision. RBB: Study conceptualization; data interpretation; manuscript revision. All authors read and approve the final manuscript.

## Pre-publication history

The pre-publication history for this paper can be accessed here:

http://www.biomedcentral.com/1471-2458/13/180/prepub
